# *In vitro* and *in vivo* efficacy of the novel oral proteasome inhibitor NNU546 in multiple myeloma

**DOI:** 10.18632/aging.104023

**Published:** 2020-11-16

**Authors:** Hui Zhou, Meng Lei, Wang Wang, Mengjie Guo, Jia Wang, Haoyang Zhang, Li Qiao, Huayun Feng, Zhaogang Liu, Lijuan Chen, Jianhao Hou, Xueyuan Wang, Chenxi Gu, Bo Zhao, Evgeny Izumchenko, Ye Yang, Yongqiang Zhu

**Affiliations:** 1 College of Life Science, Nanjing Normal University, Nanjing 210046, PR China; 2 College of Science, Nanjing Forestry University, Nanjing 210037, PR China; 3 School of Medicine and Holistic Integrative Medicine, Nanjing University of Chinese Medicine, Nanjing 210023, PR China; 4 Jiangsu Chia Tai Fenghai Pharmaceutical Co. Ltd., Nanjing 210046, PR China; 5 The 1st Affiliated Hospital of Nanjing Medical University, Nanjing 210029, PR China; 6 School of Chemistry and Materials Science, Nanjing Normal University, Nanjing 210046, PR China; 7 Department of Medicine, Section of Hematology and Oncology, University of Chicago, Chicago, IL 60637, USA; 8 The 3rd Affiliated Hospital, Nanjing University of Chinese Medicine, Nanjing 210023, PR China

**Keywords:** proteasome inhibitor, multiple myeloma, oral drug, mechanism research

## Abstract

Proteasome inhibition demonstrates highly effective impact on multiple myeloma (MM) treatment. Here, we aimed to examine anti-tumor efficiency and underlying mechanisms of a novel well tolerated orally applicable proteasome inhibitor NNU546 and its hydrolyzed pharmacologically active form NNU219. NNU219 showed more selective inhibition to proteasome catalytic subunits and less off-target effect than bortezomib *ex vivo*. Moreover, intravenous and oral administration of either NNU219 or NNU546 led to more sustained pharmacodynamic inhibitions of proteasome activities compared with bortezomib. Importantly, NNU219 exhibited potential anti-MM activity in both MM cell lines and primary samples *in vitro*. The anti-MM activity of NNU219 was associated with induction of G2/M-phase arrest and apoptosis via activation of the caspase cascade and endoplasmic reticulum stress response. Significant growth-inhibitory effects of NNU219 and NNU546 were observed in 3 different human MM xenograft mouse models. Furthermore, such observation was even found in the presence of a bone marrow microenvironment. Taken together, these findings provided the basis for clinical trial of NNU546 to determine its potential as a candidate for MM treatment.

## INTRODUCTION

The generation and degradation of intracellular proteins is essential for maintaining cellular function [1]. Protein degradation mediated via the ubiquitin-proteasome pathway (UPP) is an important mechanism regulating cellular protein homeostasis [2, 3]. UPP degrades proteins via specific attachment of ubiquitin. Proteasome substrates include damaged, misfolded and redundant proteins, and highly regulated members of critical signaling cascades [4, 5]. This system is involved in regulating cell proliferation, differentiation, apoptosis, signal transduction, antigen presentation and inflammatory responses [6]. In malignant cells, UPP is frequently dysregulated. UPP has been implicated in tumor development and multidrug resistance. A growing body of evidence suggests that proteasome inhibition is more toxic to proliferating malignant cells than to normal cells [7, 8]. The cytotoxic mechanisms include disruption of the cell cycle, triggering of apoptosis and ultimately tumor cell death [9]. Therefore, the UPP has emerged as one of the most attractive anti-cancer targets for drug design and development.

Small molecule inhibitors of the 20S proteasome are the most extensively studied proteasome inhibitors to date [10]. The first-in-class agent, bortezomib, was approved by FDA for the treatment of relapsed and/or refractory multiple myeloma (MM) or mantle cell lymphoma (MCL) [11, 12]. It is a dipeptidyl boronic acid analogue that reversibly binds the catalytic β-subunit site of the 26S proteasome in a concentration-dependent manner, thus predominantly inhibiting chymotrypsin-like (CT-L) and caspase-like (C-L) activity to a lesser degree [13]. Bortezomib has a wide range of molecular effects in MM, including inhibition of NF-κB activation and induction of apoptosis [14, 15]. Specifically, bortezomib induced the intrinsic cell death pathway mediated by activation of caspase-9 and caspase-3 and extrinsic apoptotic signaling mediated by caspase-8 and death receptors [16], as well as activation of heat shock proteins [17] and endoplasmic reticulum (ER) stress response pathways [18]. Bortezomib was also reported to influence the expression and inactivation of JNK [19] and DNA-dependent protein kinases [18]. Furthermore, bortezomib inhibited the interaction between MM cells and the bone marrow (BM) microenvironment, and blocked the transcription and secretion of associated MM cell growth factor [20]. Despite the potent anti-MM activity of bortezomib, dose-related toxicities (e.g., peripheral neurotoxicity and thrombocytopenia) limited its long-term utility in clinical practices [21]. More importantly, all of the patients who initially responded to bortezomib eventually relapsed due to development of drug resistance [22]. At present, two other proteasome inhibitors, carfilzomib and ixazomib, were launched [23]. However, treatment with these drugs was associated with a number of toxicities, including cardiotoxicity, acute renal failure, thrombocytopenia, and neutropenia [23, 24].

In order to discover more effective proteasome inhibitors as anti-cancer drugs with broad therapeutic application and better safety profiles, different series of peptidyl boronic acid derivatives were previously designed, synthesized and biologically investigated by our group [25–28]. In this study, we used lead optimization by sulfur-based technology to identify a candidate NNU546, which was a novel N-capped dipeptidyl boronic acid that immediately hydrolyzed to its biologically active form NNU219 upon exposure to aqueous solutions or plasma. NNU219 was able to selectively and irreversibly inhibit the chymotrypsin-like activity of the 20S proteasome. We demonstrated that NNU219 inhibited tumor cell proliferation, induced the G2/M-phase arrest and apoptosis, downregulated NF-κB expression, and finally triggered an ER stress response in human MM cell lines and patient-derived cells at nanomolar concentrations. Importantly, NNU219 had a favorable cytotoxicity profile in normal human lymphocytes. Finally, three distinct human MM xenograft mouse models, including a cell-line-derived xenograft model (CDX), a patient-derived xenograft model (PDX) model and a SCID-rab model were used to evaluate the *in vivo* anti-cancer activities. Our results indicated that intravenous and oral administration of NNU219 and NNU546 were promising anti-MM candidates.

## RESULTS

### NNU219 is identified as a candidate compound using sulfur-based technology

To identify novel orally bioavailable proteasome inhibitors with improved efficacy and low toxicity from our previous work [25–28], we applied a unique sulfur-based technology to design and synthesize a diverse set of peptidyl boronic acid derivatives. The incorporation of methylthio group into the chemical scaffolds was aimed to improve metabolic stability, decrease toxicity and increase oral bioavailability by increasing drug exposure (area under the curve, AUC) and prolonging half-life (t_1/2_). In the unique structural analogs, NNU219 was screened as the lead compound based on potency in proteasome inhibition and cell viability assays. Furthermore, the microsomal stabilities revealed that NNU219 had longer half-lives (t_1/2_=17-32 min) and significantly shorter clearances (CL=43-83 mL/min/kg) than bortezomib in rat, mice and human species (Supplementary Table 1). Pharmacokinetic studies of NNU219 in mice indicated a long half-life (2.08±0.991 h) and a large AUC_0-t_ (2035 h•ng•mL^-1^) following intravenous administration. After oral administration, NNU546 was absorbed rapidly (T_max_=5 min) and displayed a long half-life (2.41±0.420 h) and good oral exposure (Supplementary Table 2).

### NNU219 selectively and potently inhibits proteasome activities *in vitro*

**Figure 1:**
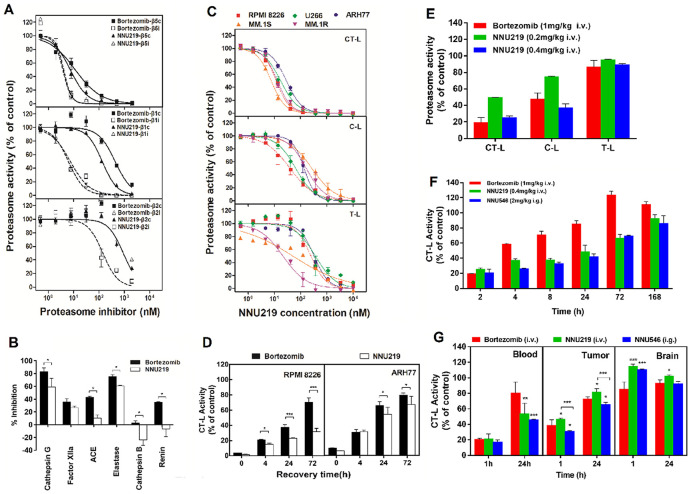
**NNU219 and bortezomib differentially affect proteasome activities *in vitro* and *in vivo*.** (**A**) *In vitro* effects of NNU219 or bortezomib on catalytic activities of the human constitutive proteasome (β1c, β2c, β5c) and the immunoproteasome (β1i, β2i, β5i). Data from at least three independent measurements were normalized to DMSO-treated controls and were presented as residual activities ± SD. (**B**) Inhibition of non-proteasomal proteases. NNU219 and bortezomib were tested at 10 μM against a panel of purified serines (Cathepsin G, Factor XIIa, Elastase), cysteine (Cathepsin B), aspartyl (Renin) and metallo (ACE) proteases. Percent inhibition was calculated based on the activities of compounds on protease subtracted with a substrate control without an enzyme. Data were presented as the mean inhibition ± SD relative to DMSO-treated controls (*, *p* < 0.5). (**C**) Selectivity of NNU219 in the active sites of human MM cell lines. MM cells were treated with various concentrations of NNU219 for 1 h and cytosolic extracts were analyzed for CT-L, C-L and T-L proteasome activities. Results were represented as percent activities of proteasome in drug-treated vs. vehicle treated cells ±SD. (**D**) Recovery of cellular proteasome activity following NNU219 or bortezomib treatment. Proteasome CT-L activity was determined in lysates prepared from RPMI 8226 (left panel) and ARH77 cells (right panel) at the indicated times following exposure to IC50 of NNU219 or bortezomib for 1 h. Mean values from three measurements are presented as the percent activity relative to control-treated cells ± SD (*, *p* < 0.5; ***, *p* < 0.001). (**E**) Proteasome active site selectivity of NNU219 *in vivo*. Mice (n=5) were treated with either NNU219 (0.2 mg/kg or 0.4 mg/kg i.v.) or bortezomib (1 mg/kg i.v.) for 1 h and whole blood was analyzed for CT-L, C-L and T-L proteasome activities. (**F**) Inhibition and recovery of proteasome activity *in vivo*. Mice (n=5) were treated with NNU219 (0.4 mg/kg i.v.), NNU546 (2 mg/kg i.g.) and bortezomib (1 mg/kg i.v.) at the indicated time-points and blood samples were analyzed for CT-L proteasome activities. The data are represented as the percent inhibition compared with vehicle treated animals from two independent experiments. (**G**) ARH77 tumor-bearing mice were administered a single dose of NNU219 (0.4 mg/kg i.v.), NNU546 (2 mg/kg i.g.) or bortezomib (1 mg/kg i.v.); mice were euthanized at 1 h and 24 h time points after treatment. Heart, brain and tumor were harvested. Protein extracts were prepared and the proteasome catalytic activity was evaluated with CT-L subunit-specific fluorescent peptide substrates. Values were presented as the mean percent activity relative to vehicle ± SD (3 mice per time-point). p values presented for bortezomib vs NNU219 or NNU546 (*, *p* < 0.5; **, *p* < 0.01; ***, *p* < 0.001). SD, standard deviation; i.v. intravenous administration; i.g. intragastric administration; CT-L, chymotrypsin-like; C-L, caspase-like; T-L, trypsin-like; MM, multiple myeloma.

The 20S proteolytic cores of constitutive proteasome (cCP) and immunoproteasome (iCP) have three different enzymatic activities, namely C-L, T-L and CT-L, which are encoded by the β1c, β2c and β5c, or β1i, β2i and β5i subunits, respectively. To determine the selectivity of NNU219 towards proteasome catalytic subunits, we first evaluated the capacity of NNU219 to inhibit the activities of purified human erythrocyte-derived cCP and iCP. The assays using the fluorogenic substrate indicated that NNU219 significantly inhibited the CT-L subunits of the constitutive proteasome (β5c) and immunoproteasome (β5i) in a dose-dependent manner (Figure 1A; *p* < 0.01) with less inhibition of the T-L and C-L activities. These results indicated the selectivity of NNU219 to inhibit the CT-L activity of the proteasome. To further understand how NNU219 and MLN2238 (Ixazomib) are bound to β5 subunit of proteasome, covalently theoretical docking was carried out and demonstrated that five strong hydrogen bonds were formed between these two small molecules with residues THR21, ALA49, GLY47 and ARG19 of β5 subunit (Supplementary Figure 1). Furthermore, additional hydrophobic interaction existed between the methylthio group of NNU219 with residue ALA49 (Supplementary Figure 1A), which was beneficial to the binding of NNU219 with the β5 subunit. However, there was no hydrophobic interaction between MLN2238 and ALA49 (Supplementary Figure 1B).

As bortezomib-induced peripheral neuropathy has been described to occur via a proteasome-independent mechanism *in vitro* and bortezomib inhibited several nonproteasomal targets *in vitro* and *in vivo* [29], the binding of NNU219 with possible non-proteasomal targets was also evaluated. NNU219 had relatively weak or no inhibitory activities against most of the non-proteasomal proteases except cathepsin G and elastase, with 50 % inhibition at 10 μM (Figure 1B), which showed that NNU219 was less potent to nonproteasomal proteins than bortezomib (Figure 1B; all *p* values < 0.05 but Factor XIIa was not significant).

It has been previously reported that the binding of proteasome inhibitors to isolated enzymes was different in the living cells [30] Therefore, it was of interest to determine the ability of NNU219 to inhibit the three subunits in MM cells. Five MM cell lines were treated with 0-10 μM NNU219 or bortezomib for 1 h and then analyzed for CT-L, C-L and T-L proteasome activities using the Proteasome-Glo cell-based assay. Incubation of MM cells with NNU219 resulted in a dose-dependent inhibition of catalytic activities of the three subunits with the activity of CT-L was inhibited to the greatest extent, which was similar to the results obtained with purified 20S proteasome (Figure 1C; *p* < 0.05). The C-L and T-L activities in the cells were inhibited to a greater extent than the inhibitory effect on the isolated enzyme (Figure 1C; *p* < 0.05). Bortezomib also exerted similar effects (Supplementary Figure 2). Although NNU219 covalently interacted with the proteasome, the dissociation of the NNU219-proteasome complex could be different from that of bortezomib. Therefore, the effect of NNU219 on the recovery of proteasome activity in RPMI 8226 and ARH77 cells was then investigated. The MM cells were treated with NNU219 for 1 h, allowed to recover in drug-free media and then CT-L activity was monitored. Our results showed that the proteasome activity in ARH77 cells treated with NNU219 recovered to 67% after 72 h, whereas the activity in RPMI 8226 cells recovered only to 31%. The proteasome activity recovery rates in NNU219-treated cells were slower than those obtained after bortezomib treatment (Figure 1D; *p* < 0.001 and *p* < 0.05 in ARH77 cells and RPMI 8226 cells, respectively). Taken together, these *in vitro* assays indicated that NNU219 is a potent and selective inhibitor of the 20S proteasome.

### NNU219 displays good pharmacodynamic profiles and oral bioactivity *in vivo*

The inhibition of 20S proteasome activity induced by NNU219 and bortezomib was further investigated *in vivo*. First, the selectivity of NNU219 and bortezomib to the active sites of the proteasome was investigated. Mice were intravenously administered with different concentrations of NNU219 (0.2 and 0.4 mg/kg) or bortezomib (1 mg/kg). Whole blood was collected after 1 h and the CT-L, C-L and T-L activities were analyzed. We found that NNU219 decreased the CT-L and C-L activities in a dose-dependent manner. Notably, while both bortezomib (1 mg/kg) and NNU219 (0.4 mg/kg) similarly inhibited CT-L (*p* < 0.001), the dosage of NNU219 used to reach this effect was substantially lower (Figure 1E). Furthermore, at doses that induced >70% inhibition of CT-L activity in the blood, the two compounds were able to inhibit the activity of C-L (*p* < 0.01). However, the two compounds did not inhibit the T-L activity (Figure 1E). These results indicated that NNU219 could selectively inhibit proteasome activities *in vivo* and were consistent with the results obtained in cell lines shown in Figure 1C.

Given that NNU219 and NNU546 had good microsomal stabilities and pharmacokinetic properties, their pharmacodynamic profiles were then investigated *in vivo* with intravenous and oral administrations. The recovery of proteasome activity in whole blood was determined at different time points after intravenous injection of NNU219 and bortezomib or oral administration of NNU546. The activities of CT-L proteasomes were reduced by up to 80% after 2 h following administration of the compounds (Figure 1F; *p* < 0.001). Proteasome activities began to recover after 4 h, almost recovered to baseline after 24 h and completely recovered after 72 h following administration of bortezomib. In contrast, much slower recovery of proteasome activities was observed following intravenous administration of NNU219 compared with bortezomib (*p* < 0.001). Furthermore, oral administration of NNU546 triggered a greater and more prolonged dose-related inhibition of proteasome activities in the whole blood (*p* < 0.001).

To evaluate the proteasome activities in tumors and normal host tissues, ARH77 MM tumor-bearing mice were intravenously injected with a single dose of NNU219 (0.4 mg/kg), bortezomib (1 mg/kg) or orally administered with NNU546 (2 mg/kg) and euthanized after 1 or 24 h post-administration based on the preliminary dose-exploratory experiment. Heart, liver, spleen, lung, kidney, brain and tumor were collected and examined for CT-L proteasome activities. The results showed that proteasome activities were significantly reduced in tumor specimens as well as blood and various normal mouse tissues (except the brain) within 1 h following administration of all three tested compounds (Figure 1G and Supplementary Figure 3; *p* < 0.01). Nevertheless, at 24 h post injection, proteasome activities gradually recovered as compared to vehicle (Figure 1G and Supplementary Figure 3). Similar proteasome activity recovery patterns were shown for bortezomib, NNU219 and NNU546. Importantly, no significant reduction in proteasome activity was detected in brain tissue at any time point for either compound (Figure 1G), which indicated that they did not cross the blood-brain barrier (BBB). Notably, oral administration of NNU546 showed much greater proteasome inhibition in blood and tumor samples compared to NNU219 and bortezomib (Figure 1G). Taken together, these data indicate that both NNU219 and NNU546 are bioavailable and selective compounds with favorable pharmacodynamic responses.

### NNU219 inhibits the proliferation of a variety of tumor cells *in vitro*

**Figure 2:**
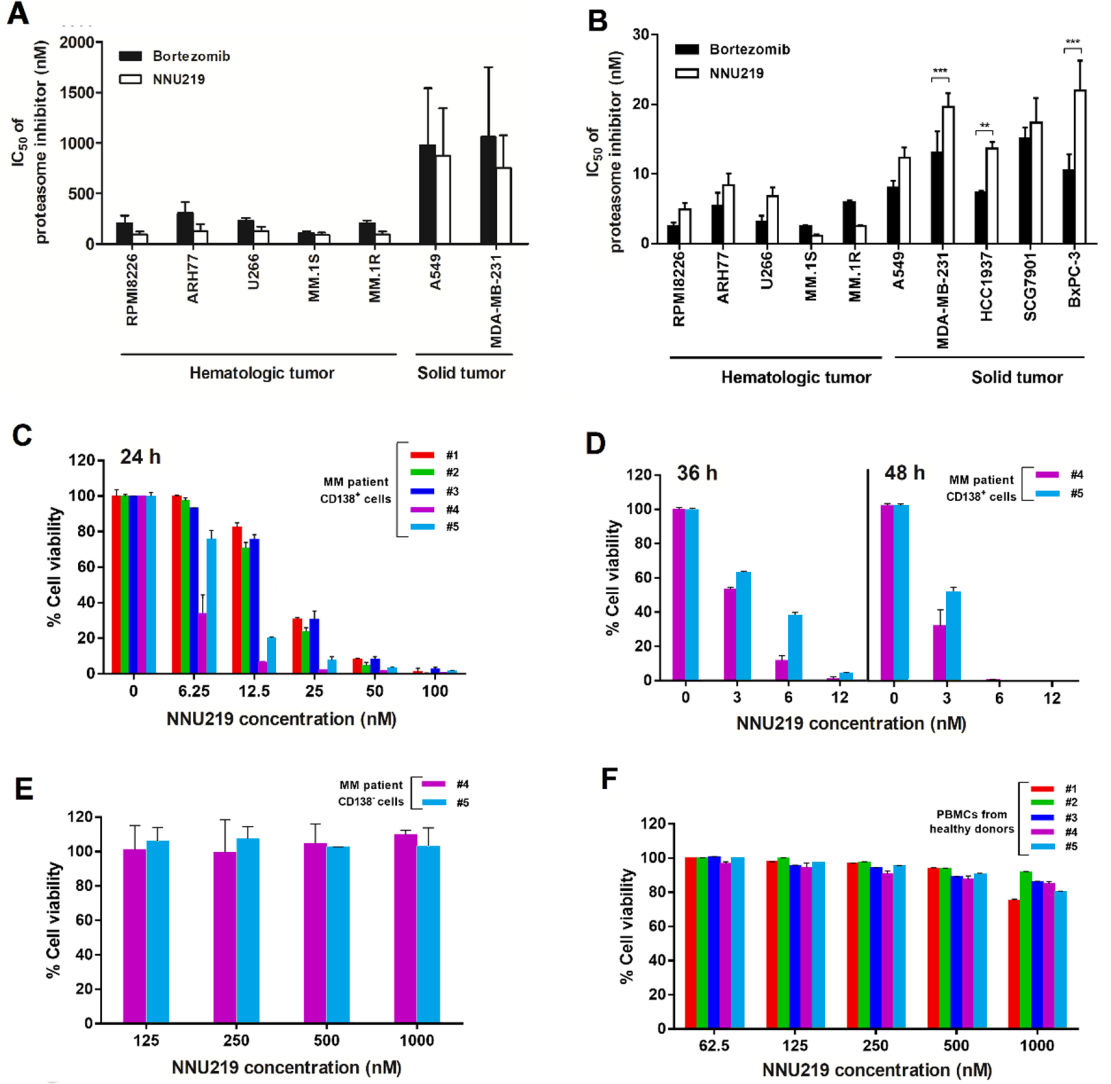
**Cytotoxicity profiles of NNU219 and bortezomib against tumor cell lines, primary myeloma cells and human PBMC.** (**A**, **B**) Hematological and solid tumor cell lines were treated with NNU219 and bortezomib. Cell viability was measured with CCK8 reagent either after 1 h of compound treatment followed by 72 h washout period (**A**) or continuous compound treatment for 72 h (**B**). The IC_50_ value as a measure the cytotoxic effects of tested compounds. Values were the mean ± SD from 3 determinations (**, *p* < 0.01; ***, *p* < 0.001). (**C**) Purified patient MM cells (CD138^+^) were treated with increasing concentrations of NNU219. The cytotoxic effects of NNU219 were determined by bioluminescent measurement of cellular ATP using Cell Titer-Glo reagent after 24 h treatment. Results were expressed as % cell viability over DMSO control. Data were presented as mean ± SD of triplicate samples (*p* < 0.001 for all patient samples). (**D**) CD138^+^ cells from MM patient no. 4 (newly diagnosed) and no. 5 (refractory) were treated with NNU219 for 36 h and 48 h, respectively. Data were presented as mean ± SD of triplicate samples (*p* < 0.001). (**E**) CD138^-^ cells from MM patient no. 4 and no. 5 were treated with NNU219 for 24 h. Data were shown as mean ± SD of triplicate samples. (**F**) PBMCs from healthy volunteers were treated with increasing concentrations of NNU219 and then analyzed for viability using Cell Titer-Glo assay. Data were shown as mean ± SD of triplicate samples.

To investigate the difference in antiproliferative activity between NNU219 and bortezomib in hematological and solid tumor cell lines, 5 hematological cell lines (MM.1S, MM.1R, U266, RPMI 8226, and ARH77) and 5 cell lines established from solid malignancies (HCC1937, MDA-MB-231, A549, SCG7901, and BxPC-3) were treated with various concentrations of the test compounds for 24-72 h and cell viability was measured using CCK-8 assay. Most previous studies evaluated the effect of proteasome inhibition on cultured cells using extended treatment periods (24-72 h), which in fact did not reflect the rapid clearance of the drug from plasma [30]. In this manuscript, the cytotoxic effects of NNU219 and bortezomib were evaluated on a panel of tumor cell lines treated for 1 h and followed by 72 h washout period. The results suggested that the two compounds had greater anti-proliferative effects against the hematological tumor cell lines than the solid ones (Figure 2A). Prolonged treatment with NNU219 and bortezomib for 72 h without washout displayed similar trend but resulted in greater cytotoxicity (Figure 2B; *p* < 0.001 for all cell lines). Suppression of recovery of proteasome activity might be responsible for the increased cytotoxicity observed with extended treatment time.

Finally, to determine the effect of NNU219 on cells isolated from patients with MM, CD138^+^ cells were purified from BM aspiration of 5 MM patients who were newly diagnosed (patients no. 2, 3 and 4) or exposed to multiple therapeutic protocols (patients no. 1 and 5 had previously received bortezomib, dexamethasone, or lenalidomide) and were treated with NNU219 for 24 h. While direct comparison between NNU219 and bortezomib in primary cultures was precluded by the relatively small number of CD138^+^ cells isolated from these patients, a significant dose-dependent decrease in viability of the cells from all MM patients was observed after NNU219 treatment (Figure 2C; *p* < 0.001 for all patient samples). Particularly, prolongation of NNU219 treatment from 36 to 48 h resulted in greater inhibition of cell viability (Figure 2D; *p* < 0.001). These results showed that the ability of NNU219 to trigger cytotoxicity was not only limited to tumor-derived MM cell lines and could be extended to primary MM cells from refractory patients including those exposed to therapy regimens with bortezomib, dexamethasone or lenalidomide. Importantly, NNU219 exhibited a favorable cytotoxicity profile toward BM-derived stromal cells (BMSCs)/CD138^-^ cells (Figure 2E). In addition, NNU219 did not significantly affect the viability of normal PBMCs from healthy volunteers, which retained >80% viability at 1,000 nM (Figure 2F), suggesting specific and selective anti-MM activity and a favorable therapeutic index for NNU219.

### NNU219 induces cell cycle arrest and apoptosis in MM cells

**Figure 3:**
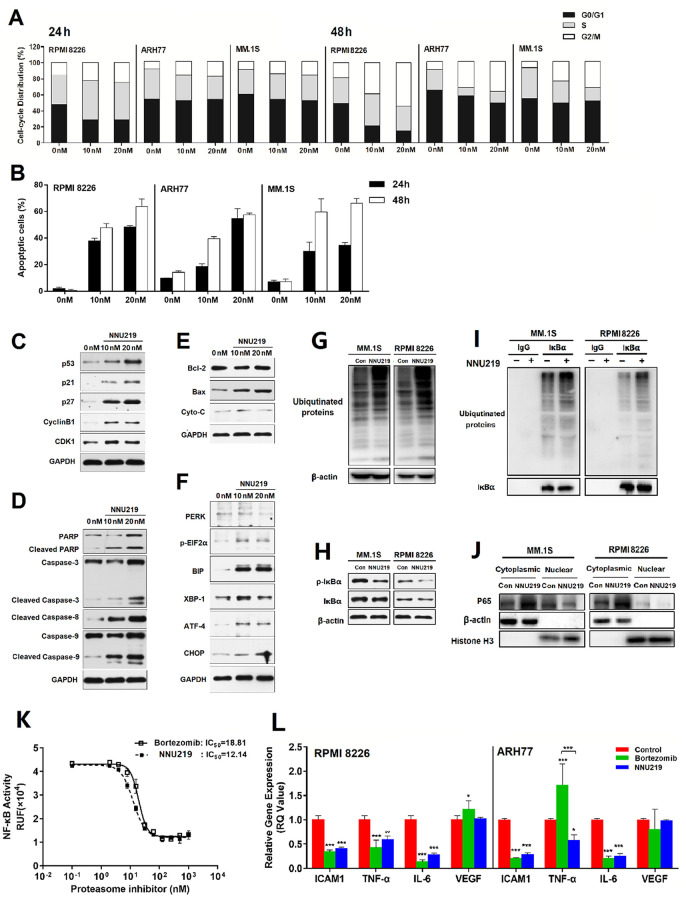
**NNU219 induces apoptotic signal transduction and modulates the NF-κB signaling pathway in human MM cell lines.** (**A**) The MM cell lines RPMI 8226, ARH77 and MM.1S were incubated with DMSO or NNU219 (10 and 20 nM) for 24 or 48 h, respectively. Cells were fixed with ethanol and stained with propidium iodide, then DNA content was determined by flow cytometry (*p* < 0.05 for all cell lines). (**B**) The MM cell lines RPMI 8226, ARH77 and MM.1S were incubated with DMSO or NNU219 (10 and 20 nM) for 24 or 48 h and induction of apoptosis was determined after annexin V-FITC/propidium iodide staining by flow cytometry. Data were presented as mean ± SD of three independent experiments (*p* < 0.001 for all cell lines). (**C**–**F**) RPMI 8226 cells were incubated with NNU219 (10 and 20 nM) for 24 h. After the incubation, cells were lysed and directly subjected to SDS-PAGE, transferred to membranes and blotted with indicated antibodies. GAPDH immunoblotting was included for protein loading control. Blots in the figures were representatives of three independent experiments. (**G**) MM1.S and RPMI 8226 cell lines were treated with 10 nM of NNU219 for 24 h. The stimulated cells were lysed and 20 μg of protein was processed for ubiquitin immunoblotting. (**H**) MM1.S and RPMI 8226 cells were treated with DMSO, 10 nM of NNU219 for 24 h and immunoblotted using anti-IκBα and anti-phospho-IκBα antibodies. (**I**) MM1.S and RPMI 8226 cell lines were treated with 10 nM of NNU219 for 24 h. Correlative proteins were immunoprecipitated from 1 mg of MM cell lysate using IκBα or IgG antibody (rabbit) and coupled to protein A/G agarose beads. The beads were washed by IP buffer and processed by immunoblotting for ubiquitin, p65 or IκBα. (**J**) MM1.S and RPMI 8226 cell line was treated with 10 nM of NNU219 for 24 h. Nuclear and cytoplasmic protein fractions were separated from the total lysate and analyzed for p65, β-actin and histone antibodies by western blot analysis. Blots in the figure were representative of three independent experiments. (**K**) The transfected NF-κB/Luciferase 293T cells were incubated with increasing concentrations of NNU219 or bortezomib for 6 h. Cells were then stimulated with 10 ng/ml of TNF-α for another 18 h. The activity of expressed luciferase was determined by using the Dual-Luciferase Reporter Assay System. (**L**) RPMI 8226 or ARH77 cell lines were treated with DMSO, IC_50_ of NNU219 or bortezomib for 12 h and harvested. Total RNA was isolated and subjected to qRT-PCR. The gene expression level of ICAM1, TNF-α, IL6 and VEGF was normalized to GAPDH using the 2^−ΔΔCT^ method. In above experiments, the control group was incubated with the same concentration of DMSO in normal culture medium. Values were expressed as mean ± SD of triplicate samples of three independent experiments (*, *p* < 0.05; **, *p* < 0.01; ***, *p* < 0.001). IL, interleukin; TNF, tumor necrosis factor; ICAM, intercellular adhesion molecule; VEGF, vascular endothelial growth factor; p-IκBα, phosphorylated inhibitor of NF-κB; Con, control; MM, multiple myeloma.

To determine the possible mechanisms responsible for the cytotoxic effects of NNU219, apoptosis and cell cycle analysis were performed after NNU219 treatment. MM cells were incubated with 0-20 nM NNU219 for 24-48 h and the cell cycle distribution was examined by flow cytometry. Compared with the untreated cells, the proportions of cells in G0/G1 and G2/M phase in the treatment groups were significantly reduced and increased, respectively (Figure 3A; *p* < 0.05). Apoptosis was then examined after Annexin V-FITC/PI fluorescence staining. Flow cytometric analysis revealed a significant increase in the apoptotic cell population after treatment with 10 and 20 nM of NNU219 for 24 h compared with the untreated cells (Figure 3B; *p* < 0.001). Incubation of MM cells with NNU219 for 48 h led to higher apoptosis than that seen at 24 h. (Figure 3B). These results indicated that NNU219 was capable of inducing apoptosis in MM cells in a time- and dose-dependent manner.

To further confirm the molecular mechanism by which NNU219 induces cytotoxic effects in cells, the expression of cell cycle-regulatory proteins and activation of caspases were examined by western blot analysis. The results showed that treatment of MM cells with NNU219 resulted in increased expressions of cyclin B1 and CDK1. Furthermore, p21, p27 and p53 were also upregulated (Figure 3C). In addition, NNU219 induced caspase-3, caspase-8, caspase-9 and PARP cleavage in dose-dependent manners. These observations indicated that NNU219 triggered mitochondria-dependent and -independent apoptotic cell death signaling pathways, leading to activation of the apoptosis in MM cells (Figure 3D). To investigate the effect of NNU219 on the mitochondrial protein family, the expression of Bax, Bcl-2 and cytochrome-C was determined following incubation of MM cells with increasing concentrations of NNU219. Although expression of the anti-apoptotic protein Bcl-2 was not changed, the levels of Bax and cytochrome-C proteins were substantially induced (Figure 3E).

Accumulation of excessive or misfolded proteins in the ER triggers the unfolded protein response (UPR) [23]. Previous studies indicated that induction of ER stress in MM cells was an effective anti-cancer strategy and attributed to one of the mechanisms of cytotoxicity in MM cells [23, 31]. Therefore, the role of NNU219 in regulating the expression of UPR components in MM cells was then investigated, revealing that NNU219 caused activation of the PERK-mediated ER stress signal. As shown in Figure 3F, NNU219 induced elevation of BIP and XBP-1 together with triggered phosphorylation of EIF2α, further leading to expression of transcription factor ATF-4 and pro-apoptotic signaling molecule CHOP. These results indicate that NNU219 induces cell death via induction of UPR-dependent ER stress.

As inhibition of proteasome activity leads to blockade of the proteasome-dependent polyubiquitination protein degradation pathway and thereby triggers apoptosis [22], MM cells were treated with NNU219 and then the protein lysates were subjected to western blot analysis with a ubiquitin-specific monoclonal antibody (Figure 3G). The results revealed that incubation with NNU219 resulted in marked accumulation of ubiquitinated proteins as a result of inhibited proteasome function, which may induce activation of multiple pro-apoptotic signaling axes [22, 30].

### NNU219 targets NF-κB activity

It was previously reported that one of the major mechanisms underlying the treatment of MM by proteasome inhibitors is blockade of the NF-κB signaling [6, 32]. NF-κB mediated immune and inflammatory responses and was thought to be associated with proliferation, survival and drug resistance in MM cells [23]. Therefore, as a next step we examined whether NNU219 could reduce NF-κB activity in MM cells. The results demonstrated that NNU219 substantially blocked the phosphorylation of IκBα (Figure 3H). Inhibition of IκBα phosphorylation indicates reduced degradation of this protein and subsequently more binding to NF-κB, thereby blocking the NF-κB signaling pathway. Given the effects of NNU219 on NF-κB pathway regulation, the role of NNU219 in the ubiquitination of IκBα was also investigated. As presented in Figure 3I, NNU219 promoted the accumulation of IκBα ubiquitination in MM cells. Moreover, immunoblotting analysis of cytoplasmic and nuclear extracts demonstrated that nuclear p65 level was substantially reduced by NNU219 (Figure 3J). Next, NF-κB/Luc 293T cells were used to detect NF-κB activity. The 293T cell line was transfected with luciferase reporter vector under the control of NF-κB-responsive element. NF-κB was rapidly activated by stimulation with TNF-α, inducing an increase in the luciferase activity. However, pre-treatment of NF-κB/Luc 293T cells with NNU219 led to a significant reduction of luciferase activity in a time-dependent manner (Figure 3K, *p* < 0.001). Several studies previously suggested that adhesion of MM cells to BMSCs triggered NF-κB-mediated transcription and cytokine secretion, which was associated with growth and survival of MM cells, including the production of cytokines (IL-6, IL-1-β, TNF-α), cell adhesion molecules (ICAM), vascular cell adhesion molecule (VCAM) and the proangiogenic factor (VEGF)c. Therefore, the expression levels of these genes were tested in RPMI 8226 and ARH77 cells after either NNU219 or bortezomib treatment. The results showed that NNU219 inhibited the expression of the cytokines, while no significant changes in the expression of VEGF were observed. However, treatment with bortezomib resulted in upregulation of VEGF expression in RPMI 8226 cells and of TNF-α in ARH77 cell line (Figure 3L). Collectively, these results indicated that NNU219 targets NF-κB and related cytokine secretion similarly to, or even better than bortezomib, albeit in lower concentration.

### NNU219 inhibits tumor growth in human MM cell xenograft mouse models

**Figure 4:**
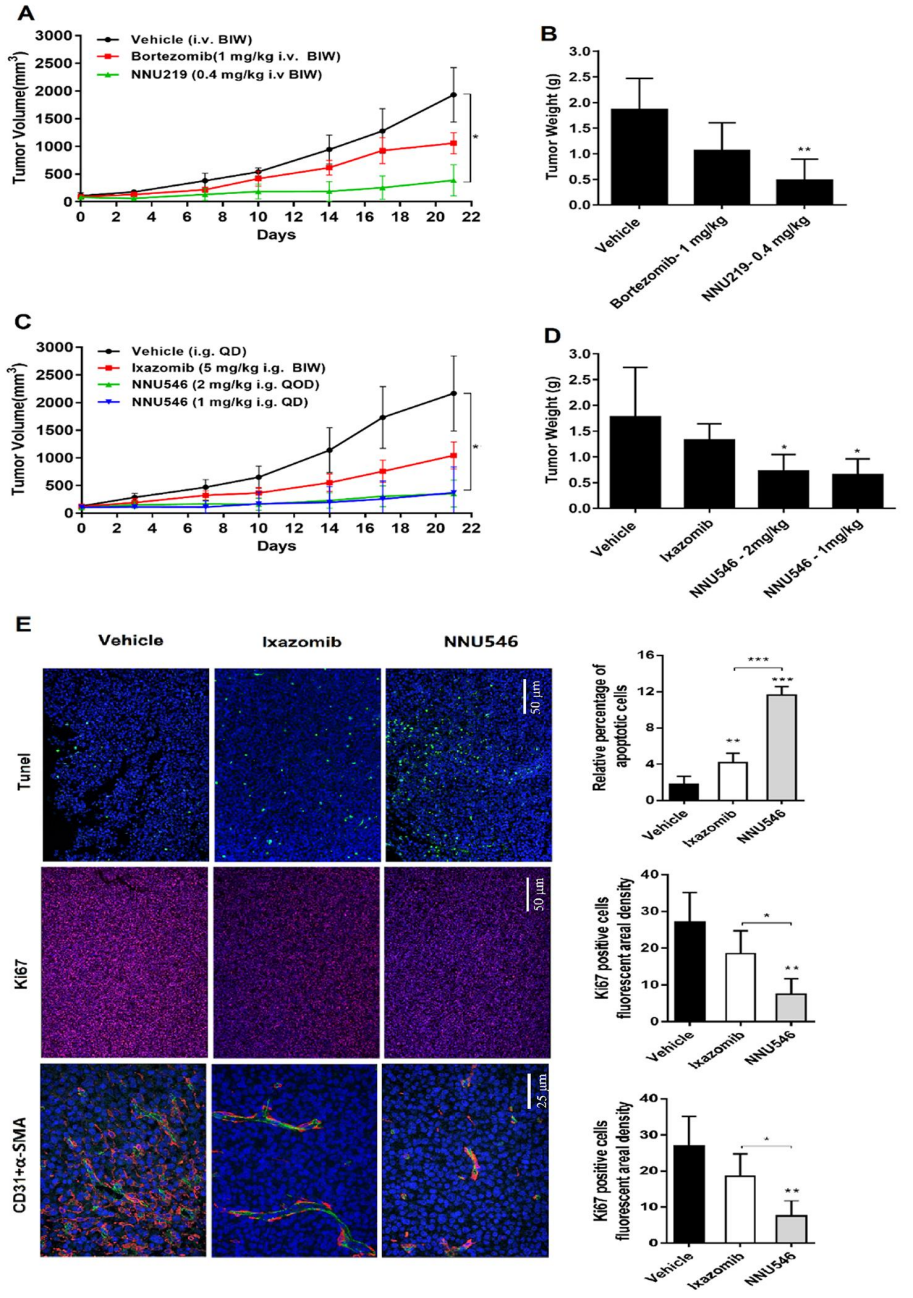
**Effects of NNU219, NNU546, bortezomib and ixazomib on the growth of ARH77 xenograft model established in nude mice.** (**A**) 5×10^6^ of ARH77 cells were subcutaneously inoculated into the right flank of nude mice. When the mean tumor volume reached 100-150 mm^3^, mice were randomized into vehicle group (1% DMSO and 5% HPβCD) and treatment group (bortezomib, 1 mg/kg or NNU219, 0.4 mg/kg), intravenously administered twice weekly for 3 weeks. The measurement was performed using a caliper. Data were presented as mean tumor volume ± SD (n=5; *, *p* < 0.05). (**B**) Average tumor weight of mice in the vehicle and treatment groups. Data were showed as mean ± SD (**, *p* < 0.01). (**C**) Tumor-bearing mice were orally treated with vehicle, ixazomib (5 mg/kg, BIW) or NNU546 (1 mg/kg, QD or 2 mg/kg, QOD) schedule for 3 weeks. Data were presented as mean tumor volume ± SD (n=5; *, *p* < 0.05). (D) Average tumor weight of mice in the vehicle and treatment groups. Data were showed as mean ± SD (*, *p* < 0.05). (**E**) Left, tumor sections of untreated, Ixazomib or NNU546 treated mice were subjected to immunostaining for apoptosis (TUNEL, green color), proliferation marker Ki-67 (red color) and angiogenesis markers CD31 (green color) and α-SMA (red color) and detected using confocal microscopy (PerkinElmer UltraVIEW Vox, magnification, ×200 or ×400, nine continual fields put together). Right, the tissue sections were screened under a low-power field, and five fields were selected. Each field was analyzed separately to obtain the fraction of apoptotic cells, ki67 positive cells fluorescent areal density and microvessel density. The data is presented as mean ± SD across the vision fields (*, *p* < 0.05; **, *p* < 0.01; ***, *p* < 0.001). SD, standard deviation; TUNEL, terminal deoxynucleotidyl transferase deoxyuridinetriphosphate nick end labelling; SMA, smooth muscle actin.

NNU219 induced MM cells apoptosis *in vitro* and had good pharmacodynamic profiles *in vivo*. Therefore, we next evaluated the anti-tumor efficacy of NNU219 using 3 different xenograft mouse models (Figure 4). Both NNU219 and NNU546 dosing schedules were tolerated in the tumor-bearing animals, resulting in weight loss of <10% (data not shown). The bortezomib and ixazomib dosing schedules were determined to be the maximum tolerated dose (MTD) in this mouse strain [3, 12]. First, ARH77 tumor-bearing mice were intravenously treated with bortezomib (1 mg/kg, BIW), NNU219 (0.4 mg/kg, BIW) or vehicle (BIW), respectively. As shown in Figure 4A and 4B, administration of either NNU219 or bortezomib significantly reduced the tumor burden compared with vehicle group (*p* < 0.05). Furthermore, NNU219 exhibited a relatively higher efficacy than bortezomib in reducing the tumor volume (75.3 vs. 24.9%). For oral administrations (i.g.), the anti-tumor effect of NNU546 (1 mg/kg, QD or 2 mg/kg, QOD) was investigated along with ixazomib (5 mg/kg, BIW) as the positive control. The daily dosing schedules of NNU546 was more intensive than the preclinical dosing schedule for ixazomib, preventing full recovery of proteasome activity between doses. Animals were continuously treated for 3 weeks and the tumor volume was measured. A dose-dependent tumor growth inhibition was achieved by NNU546 with 1 and 2 mg/kg, leading to reduction in tumor volume by 74.6% and 83.9%, respectively (Figure 4C, 4D; *p* < 0.05).

Treatment with ixazomib also reduced tumor progression by 36.3%, but less than the effect of NNU546. Importantly, no weight loss, diarrhea, hair loss, or neurological symptoms were observed in NNU546-treated mice, even if mice were treated daily with 1 mg/kg dose (data not shown), which may indicate that NNU546 has greater tolerability and less toxic *in vivo*.

In addition, paraffin-embedded sections of tumors from xenograft mice were subjected to immunofluorescence (IF) staining. The results are shown in Figure 4E. TUNEL positive cells (green color, an indication of cell apoptosis) were increased in tumor sections of NNU546-treated mice compared with those of the vehicle-treated counterparts. In parallel, the proliferation marker Ki-67 (red color) was also significantly decreased. These results confirmed the potent apoptosis-inducing effect of NNU546 in MM cells *in vivo*, which was consistent with the *in vitro* data. MM cell growth was previously reported to be associated with angiogenesis [22, 31]. Therefore, the anti-angiogenic activity of NNU546 was then evaluated by using two distinct markers of angiogenesis, CD31 and α-SMA. As shown in Figure 4E, treatment with NNU546 reduced the density of CD31 (green color), α-SMA (red color) and the number of endothelial cells and surrounding pericytes in tumor tissues, further confirming the *in vivo* anti-tumor and anti-angiogenesis activities of NNU546 in MM.

**Figure 5:**
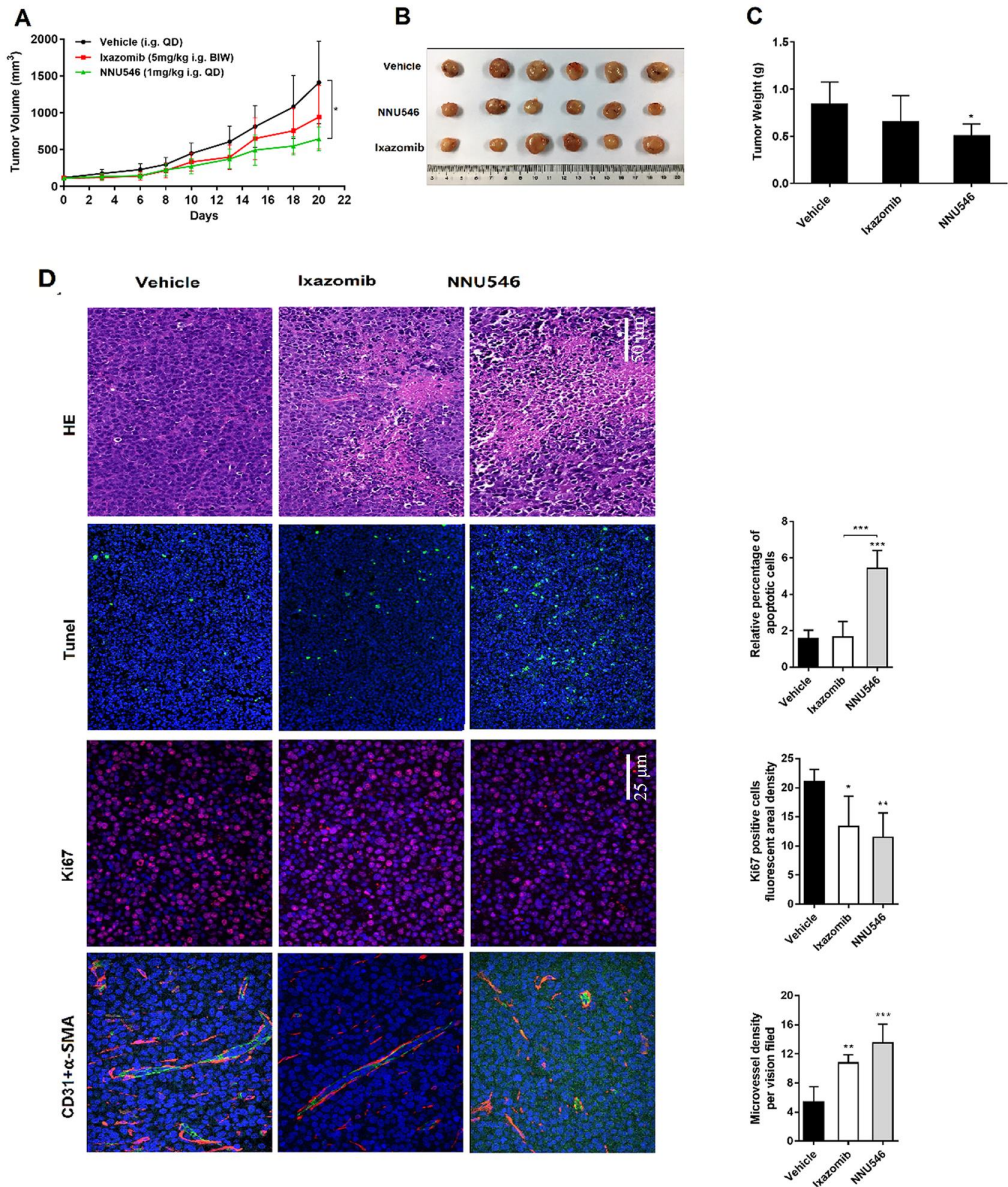
**Effects of NNU546 and ixazomib on tumor growth in PDX model.** (**A**) Relative tumor growth of PDX models treated orally with vehicle, ixazomib (5 mg/kg, BIW) or NNU546 (1 mg/kg, QD) scheduled for 3 weeks. Data were presented as mean ± SD (n=6;, *p* < 0.05). (**B**) Differences in tumor size for the vehicle and treatment groups. The tumors were resected from the NCG mice at the end of the experiment. (**C**) Average tumor weight in the vehicle and treatment groups. Data were shown as mean ± SD (, *p* < 0.05). (**D**) Left, H&E and immunostaining of tumor sections of vehicle, ixazomib and NNU546 treated mice, including apoptosis-associated TUNEL (green color), proliferation marker Ki-67 (red color) and angiogenesis markers CD31 (green color) and α-SMA (red color), detected using confocal microscopy (PerkinElmer UltraVIEW Vox, magnification, ×200 or ×400, nine continual fields put together). Right, the tissue sections were screened under a low-power field, and five fields were selected. Each field was analyzed separately to obtain the fraction of apoptotic cells, ki67 positive cells fluorescent areal density and microvessel density. The data is presented as mean ± SD per across the fields, (*, *p* < 0.05; **, *p* < 0.01; ***, *p* < 0.001). TUNEL, terminal deoxynucleotidyl transferase deoxyuridinetriphosphate nick end labelling; PDX, patient-derived xenograft; SMA, smooth muscle actin; SD, standard deviation.

To further evaluate the therapeutic efficacy of NNU546, patient-derived primary human MM xenografts implanted in NCG mice were established. Compared with the control group, treatment with NNU546 (1 mg/kg, i.g., QD) significantly inhibited the tumor growth (46.1% of reduction of tumor volume) (Figure 5A–5C; *p* < 0.05). In comparison, ixazomib (5 mg/kg, i.g., BIW) had a less potent effect to induce tumor regression (30.5% of reduction of tumor volume). Once the administrations were completed, the mice were euthanized and tumor sections were examined by H&E staining. We found that large areas of necrosis with abundant nuclear debris appeared in xenograft mice treated with NNU546. In addition, NNU546 markedly reduced the expression of Ki67, the density of CD31 and α-SMA in blood vessels and increased the apoptosis of MM cells detected by IF assay (Figure 5D).

**Figure 6:**
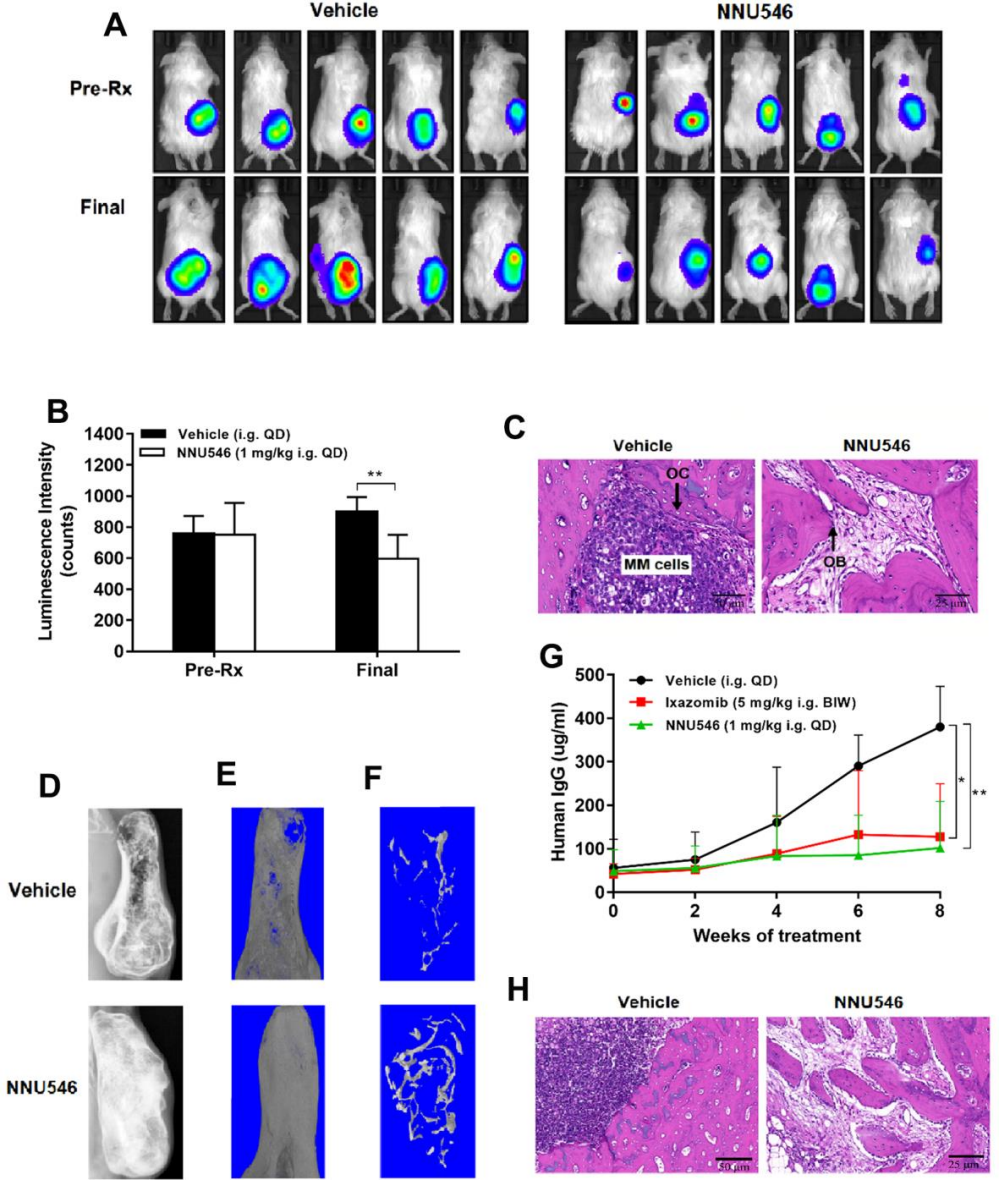
**NNU546 inhibits the growth of human MM cells in SCID-rab mouse model.** (**A**) Rabbit bone grafts were subcutaneously implanted into SCID mice. After four weeks, 5×10^6^ of RPMI 8226 cells with luciferase expression were injected directly into the rabbit bone in SCID mice. After establishment of this *in vivo* model, mice were orally administered with either vehicle or NNU546 (1 mg/kg, QD). Representative imaging of the live animals demonstrated the luciferase expression prior to treatment (Pre-Rx) and at the end of the experiment (Final) in mice. (**B**) Luminescence intensity was quantified using living animal imaging of mice prior to treatment (Pre-Rx) and at the end of the experiment (Final). Data were expressed as mean ± SD (n=5; **, *p* < 0.01). (**C**) H&E staining of histological sections of myelomatous bones engrafted with myeloma cells (×200 or ×400 original magnification). Increased myeloma cell infiltration and osteoclast activity were noted in control group. In contrast, myelomatous bone from a host treated with NNU546 had no apparent myeloma cells but possessed increased number of trabecular bone and osteoblast. (**D**) SCID-rab mice engrafted with myeloma cells from MM patients were treated with vehicle, ixazomib (5 mg/kg, BIW) or NNU546 (1 mg/kg, QD). X-radiographs of myelomatous bones engrafted with myeloma cells from MM patients were performed at the end of the experiment. (**E**, **F**) Representative microCT images of bone grafts excised from vehicle-treated animals and mice that had received NNU546 for 8 weeks. (**G**) Effect of therapy on myeloma growth assessed by human IgG levels in the mouse serum measured by ELISA. Data were shown as mean ± SD (n=5; *, *p* < 0.05; **, *p* < 0.01). (**H**) H&E staining of histological sections of myelomatous bones engrafted with cells from MM patients (original magnification, ×200 or ×400 original magnification).

The BM microenvironment of MM hosts and related cytokine secretion had been reported to be associated with proliferation, survival, migration and drug resistance in MM cells [33]. In this manuscript, two different SCID-rab mouse models were used to investigate the potency of NNU546 against MM in the presence of the BM microenvironment, which were similar to the SCID-hu model [34]. In this SCID-rab model, RPMI 8226-Luc cells expressing a luciferase reporter gene were directly injected into rabbit bone chips that were implanted subcutaneously in SCID mice and MM tumor growth was tracked by quantitative Xenogen imaging in living animals. A robust anti-tumor response was observed in mice receiving an oral dose of 1 mg/kg of NNU546 once daily compared with vehicle (Figure 6A, 6B; *p* < 0.01). The H&E staining of histological sections of the implanted bone of MM-bearing SCID-rab mice revealed massive myeloma cell infiltration and increased osteoclast activity in control group (Figure 6C). In contrast, implanted bone of a host treated with NNU546 showed no obvious myeloma cells survival, while an increased number of trabecular bone and osteoblasts were observed.

We next assessed the level of circulating human monoclonal IgG in mouse serum as a marker for monitoring the tumor burden in SCID-rab models. The therapeutic efficacy of drugs against MM-induced bone disease was evaluated by measuring the implanted bone mineral density (BMD) changes by the microCT system. The association between the anti-myeloma and bone anabolic effect of NNU546 was visualized by X-radiographs. As shown in Figure 6D, osteolytic bone lesions were evident in implanted bones of control hosts at the end of the experiment. MicroCT imaging of bone grafts revealed that great portions of the bone graft in the control group were eroded (Figure 6E) and that trabecular bone was reduced (Figure 6F). Tumor burden assessed by the human monoclonal IgG content in serum showed that the decrease of IgG levels in the NNU546 group paralleled the significant reduction of tumor volume compared with the vehicle group (Figure 6G; p < 0.01). Moreover, in contrast to control animals, the structure of the bone grafts in mice that had received NNU546 therapy was well preserved. In addition, the BMD of the implanted bone treated with NNU546 was higher than that of the control (Supplementary Figure 4A). The histomorphometric analysis revealed that the bone volume/total volume, trabecular thickness and trabecular number in myelomatous bones treated with NNU546 were all increased (Supplementary Figure 4B–4D), indicating that NNU546 reduced bone loss in the grafts. Histological examination of bone grafts treated with NNU546 revealed that the osteoblasts were increased and the multinucleated osteoclasts were reduced compared with those in the vehicle group (Figure 6H).

In summary, the results from 3 different MM xenograft mouse models demonstrated the potent *in vivo* antitumor activity of NNU219/NNU546 at well-tolerated doses. The results obtained with the SCID-rab model provided *in vivo* evidence for the ability of NNU219/NNU546 to trigger apoptosis of tumor cells even in the presence of the BM microenvironment.

## DISCUSSION

Proteasome inhibition is a highly effective mechanism of action in MM treatment. The first-generation proteasome inhibitor bortezomib was approved for the treatment of MM and provided a great benefit for patients. However, despite significant improvements in the survival of MM patients, frequent administration led to development of drug resistance and toxic effects, particularly peripheral neurotoxicity (PN). Carfilzomib, the second-generation proteasome inhibitor, was associated with a low incidence of PN, but serious adverse events (SAEs) and was intravenously administered [23, 35]. As most patients become resistant against these drugs or can no longer tolerate treatment due to specific side effects such as neurotoxicity (bortezomib) or cardiotoxicity (carfilzomib), there is an urgent need to develop a novel well tolerated orally applicable proteasome inhibitor effective in bortezomib and/or carfilzomib resistant patients. In this study, a novel orally administered bioactive dipeptidyl boronic acid candidate, NNU546, was demonstrated to have potent *in vitro* anti-cancer activity and *in vivo* efficacy in different xenograft mouse models of MM.

The enzymatic activities of NNU219 (a biologically active form of NNU546 to which it hydrolyzes upon exposure to aqueous solutions or plasma) against the proteasome were evaluated. NNU219 significantly inhibited the CT-L subunits of constitutive proteasome and immunoproteasome in a dose-dependent manner with minimal inhibition of T-L and C-L activities. Theoretical docking results revealed the binding mode of NNU219 with β5 subunit (five strong hydrogen bonds were formed except an additional hydrophobic interaction). This indicates that NNU219 was able to effectively bind proteasome. Recently, it was demonstrated that hematologically derived cells expressed the subunits β5i and β5c, and inhibition of only one subunit did not alter cell viabilities [36]. Inhibition of CT-L activity represents a valid and successful anti-tumor strategy in hematological tumors without causing cytotoxicity in non-transformed cells [37]. Similar results on the selectivity among the three subunits were obtained in different MM cell lines and mice. It revealed that NNU219 inhibited all three proteasome activities in MM cell lines, which was different from the results of the enzymatic assay. The differences between the proteasome and cells were possibly due to the specific environment in the cell, including metabolism of compounds, protein-protein interactions, post-translational modifications and asymmetric intracellular localization of compounds [38]. Furthermore, the activity of proteasome in cell slowly recovered after drug exposure, which was possibly caused by the induction of mRNA transcription and *de novo* synthesis of individual subunits and their further transformation into a new proteasome [30].

In the present study, it was demonstrated for the first time that NNU219 markedly inhibited cell growth in various MM cell lines, including those that were sensitive and resistant to conventional therapies with various cytogenetic backgrounds. Although the activity of NNU219 varied among different MM cell lines, it was more active against MM.1S and MM.1R cells with wild-type p53. P53 and its downstream genes encoding the proteins p21 and p27 are endogenous cyclin-dependent protein kinase inhibitors, and suppressed cell cycle progression [22]. Our study indicated that NNU219 induced the expression of p53, p21 and p27 in MM cells. Treatment with NNU219 also caused upregulation of the expressions of cyclin B1 and CDK1. As the degradation of these proteins is mediated by the ubiquitin-proteasome pathway, it was speculated that NNU219 might block proteasome activity, resulting in inhibition of the degradation of cyclin B1 and CDK1, which leads to their accumulation. As it was not possible to normally activate CDK1, cells were not able to enter mitosis and were arrested in G2/M phase of the cell cycle, resulting in induction of apoptosis. The flow-cytometry results further confirmed that inhibition of cell growth was attributed to induction of apoptosis. NNU219 induced apoptosis by activation of the caspase cascades, which was reflected by increased cleavage of caspase-9, caspase-8, caspase-3 and PARP, suggesting that NNU219 triggered the intrinsic and extrinsic pathways of apoptosis. Furthermore, NNU219 caused accumulation of proteasome substrates, including polyubiquitinated proteins, which then led to activation of UPR, as evidenced by the activation of ATF4 and XBP1, and the induction of BIP and CHOP expression. Of note, the present results suggested that NNU219 reduced the viability of tumor cells of patients with primary MM who exhibited disease progression after various treatments without affecting the viability of normal PBMCs. Results obtained from multiple cell lines showed that proliferating malignant cells were affected by NNU219 due to the accumulation of damaged proteins at a much higher rate than that in normal quiescent cells [39, 40].

NF-κB is a key regulator of growth and survival of MM cells. It is chronically active in MM cells and may be induced by cytokines, prosurvival factors, chemokines and contact with the components of microenvironment. Many studies have demonstrated that bortezomib prevented IκBα from degradation by proteasome, thus inhibiting nuclear translocation of NF-κB, cytokine circuits and the survival advantage for MM cells conferred by the BM microenvironment [6, 34, 41]. Previous studies suggested that the anti-cancer activity of bortezomib in MM might be attributed to the inhibition of inducible NF-κB activity [6, 32]. However, recent evidence suggested a paradigm shift in this mechanism [42]. Bortezomib enhanced constitutive NF-κB activity via activation of the canonical pathway, which suggested that bortezomib-induced cytotoxicity might not be completely ascribed to the inhibition of canonical NF-κB activity in MM [23, 31]. *In vitro* studies indicated that NNU219 inhibited the NF-κB activation through promoting the accumulation of ubiquitination. In addition, NNU219 caused downregulation of the expression of several downstream effectors of NF-κB. The expression of these NF-κB-mediated genes is known to affect growth factors, cell adhesion, angiogenesis and resistance to chemotherapeutic agents and radiotherapy [6].

By testing *in vivo* pharmacokinetic and pharmacodynamic characteristics, NNU546 was identified as an orally bioavailable candidate. The biodistribution and inhibition profile of proteasome activities of NNU546 *in vivo* was investigated. Oral administration of NNU546 exerted significant proteasome inhibition in whole blood at 1 h, which was recoverable at 168 h, whereas bortezomib inhibited proteasome activity with only minimal or no inhibition at 48 h. In all tissues except the brain, recovery of the proteasome activity from NNU219/NNU546-mediated inhibition was comparable to that of bortezomib and the recovery of proteasome activity in the tissues was faster than in the blood, indicating that new proteasome synthesis had a dominant role in the recovery of proteasome activity in tissues other than in whole blood. This might be due to the inability of mature erythrocytes to restore their activity by generating new proteasomes, whereas the recovery of proteasome activity in whole blood depended on the production of new erythrocytes.

To confirm the *in vitro* results, the present study also employed several types of xenograft mouse models derived from human MM to investigate the efficacy of the compounds *in vivo*. The results indicated that NNU219/NNU546 significantly inhibited tumor growth and prolonged the survival of mice in all MM animal models. The results obtained using the MM xenograft models indicated that the significant and sustained anti-tumor efficacies achieved by intravenous or oral administration of NNU219/NNU546 were associated with the pharmacodynamics of the drugs. Although xenografts derived from human tumor cell lines allowed for rapid evaluation and prioritization of potential drug candidates for progression to clinical trials, these models did not fully reflect the biology and heterogeneity of their human disease counterparts. To reduce intra-tumoral heterogeneity, PDX models were established in mice to more accurately model the characteristics of human tumors. The results indicated that treatment with NNU546 in PDX models significantly inhibited the tumor growth compared with the control group. Moreover, no significant toxicity was observed in NNU546 treatment group. The potent anti-MM activity of NNU546 *in vivo* was confirmed by IF analysis of tumor sections of control and NNU546 treated mice. The results suggested that NNU546 induced MM cell apoptosis (TUNEL) and decreased the expression of MM cell proliferation (Ki-67) and angiogenesis (CD31 and α-SMA). The SCID-rab mouse model reflected the interactions between the human BM microenvironment and human MM cells. Importantly, our data provided evidence that NNU546 directly targeted MM cells and overcame the cytoprotective effects of the BM microenvironment on MM cells.

In conclusion, our results demonstrated that compound NNU546 was a novel orally active candidate and being investigated as a new drug. We believe that this new candidate compound will benefit for the MM patients for better curative effect and less side effects.

## MATERIALS AND METHODS

### Cell cultures

The human myeloma cell lines MM.1S, MM.1R, U266, RPMI 8226, ARH77, HCC1937, MDA-MB-231, A549, SCG7901 and BxPC-3 (all from the American Type Culture Collection, USA) were cultured in RPMI1640 medium supplemented with 10% fetal bovine serum (FBS) and 1% sodium pyruvate. All cell lines were routinely maintained at 37 °C in 5% CO_2_. After Ficoll-Hypaque density gradient centrifugation, mononuclear cells from patients’ BM aspirates were subjected to CD138 (Syndecan-1) purification using Micro Beads and the Auto MACS magnetic cell sorter (Miltenyi Biotech, Bergisch Gladbach, Germany) according to the manufacturer’s protocol. CD138-positive cells with a purity of > 95% verified by flow cytometric analysis were used for further experiments. Peripheral blood mononuclear cells (PBMCs) were obtained from healthy individuals after Ficoll-Hypaque density separation as previously described [33]. Informed consent was obtained from all patients and volunteers in accordance with the Declaration of Helsinki. The ethical number is 2013-SRFA-067.

### Enzyme activities and inhibition assays

Inhibition of CT-L, C-L, and trypsin-like (T-L) activities of 20S proteasome was assessed using purified human erythrocyte-derived constitutive 20S proteasomes (cCP) and 20S immunoproteasome (iCP; Boston Biochem, Cambridge, MA, USA), as previously described [43, 44]. Standard substrate-based assays were initiated by enzyme in the presence of 10 μM of the test compounds for the non-proteasome protease panel assay (GenScript, Nanjing, China) as previously described [29].

### Docking studies

To investigate the binding mode of NNU219 with β5 subunit of proteasome, *in silico* modelling of docking was carried out. The covalent docking was performed using the Glide module of Schröedinger software (LLC, New York, NY, USA, 2015). The THR1 residue of β5 subunit of proteasome (PDB ID: 4R67) was covalently anchored with the boronic acid fragments of NNU219 and MLN2238, respectively. And other groups of the molecules were docked with the residues of the proteasome.

### Intracellular and *in vivo* proteasome activity assays

For the cellular proteasome activity assays, cells were treated with concentrations of compounds (0-10 μM) for 1 h at 37 °C and the CT-L, C-L and T-L activities of proteasome were evaluated by using the Proteasome-Glo assay reagents (Promega Corp. Madison, WI, USA) according to the manufacturer's protocol. For determination of recovery of cellular proteasome activity, cells were treated with the compounds for 1 h and then washed thrice in PBS to remove the compounds. Cells were incubated in fresh medium for an additional 4, 24 or 72 h at 37 °C. To investigate the *in vivo* proteasome activities, female beige nude xid (BNX) mice (5-7 weeks old; Shanghai Sippr-BK laboratory Animal Co. Ltd.) were intravenously administered with a single dose of NNU219 (0.2 or 0.4 mg/kg) or bortezomib (1 mg/kg), respectively. Blood samples were collected after 1 h, and whole blood cells were then analyzed for proteasome activity as previously described [45].

### Cell viability, cell cycle and apoptosis assays

Cell viability assay was determined using a Cell Counting Kit-8 (CCK-8; Dojindo Laboratories, Kumamoto, Japan). In brief, cancer cells were suspended and seeded in the culture medium in a 96-well plate. After 24 h, cells were treated with the compounds at 37 °C for 1 h or 72 h followed by washing thrice with PBS and further incubation in fresh medium for an additional 72 h. The CCK8 reagent was added to each well and the plates were incubated at 37 °C for another 1-2 h. The absorbance was measured with a microplate reader (CLARIOstar; BMG Labtech) at 450 nm. Cell viability was also assessed by exclusion of trypan-blue staining. The viabilities of CD138-positive cells and PBMCs were assessed using the Cell Titer-Glo Luminescent Cell Viability Assay kit (Promega, Corp., Madison, WI, USA) with cells plated and processed as described above. Cellular DNA content was quantified using a propidium iodide (PI) staining assay kit (BD Biosciences, San Diego, CA, USA). The percentage of apoptotic cells was detected using the Annexin V/PI staining assay kit (BD Biosciences, San Diego, CA, USA). Cell cycle and apoptosis were analyzed by flow cytometry (FACSCalibur, BD Biosciences).

### Western blot analysis

Western blot analysis was performed as previously described [46]. In brief, equal amounts of protein were prepared, resolved on 15% SDS-PAGE and transferred onto PVDF membranes. Membranes were blocked by incubation in 5% nonfat dry milk in Tris-buffered saline containing Tween-20 (TBST) for 1 h, and probed with specific antibodies to ubiquitinated proteins (#3933, Cell Signaling Technology, MA, USA), caspase-3 (#9662, Cell Signaling Technology, MA, USA), caspase-8 (#4927, Cell Signaling Technology, MA, USA), caspase-9 (#9502, Cell Signaling Technology, MA, USA), BIP (#3183, Cell Signaling Technology, MA, USA), phosphorylated (p)-eukaryotic translation initiator factor (EIF)2α (#9721, Cell Signaling Technology, MA, USA), X-box binding protein (XBP)1 (#83418, Cell Signaling Technology, MA, USA), activating transcription factor (ATF)4 (#11815, Cell Signaling Technology, MA, USA), CHOP (#2895, Cell Signaling Technology, MA, USA), inhibitor of NF-κB (IκBα; #9242, Cell Signaling Technology, MA, USA), p-IκBα (Ser32; #2859, Cell Signaling Technology, MA, USA), p65 (#6956, Cell Signaling Technology, MA, USA), β-actin (#4970, Cell Signaling Technology, MA, USA), poly (ADP ribose) polymerase (PARP; #551024, BD Bioscience Pharmingen, San Diego, CA, USA), p53 (sc-47698, Santa Cruz Biotechnology, Inc., CA, USA), p21 (sc-24559, Santa Cruz Biotechnology, Inc., CA, USA), Cyclin B1 (sc-70898, Santa Cruz Biotechnology, Inc., CA, USA), cyclin-dependent kinase (CDK1; sc-53219, Santa Cruz Biotechnology, Inc., CA, USA), PERK (sc-377400, Santa Cruz Biotechnology, Inc., CA, USA) and GAPDH (sc-69778, Santa Cruz Biotechnology, Inc., CA, USA) at 4 °C for 12 h, respectively. After washing with TBST, membranes were incubated with a peroxidase-conjugated secondary antibody (Bioworld, Nanjing, China) at room temperature for 1 h. Blots were visualized using enhanced chemiluminescence reagents (Bioworld, Nanjing, China). The experiments are performed for three times.

### Co-immunoprecipitation (Co-IP) and ubiquitination

Co-IP was performed as previously described [47]. In brief, the MM cell lines MM.1S and RPMI 8226 were treated with 10 nM of NNU219 for 24 h. The control group was incubated in the same concentration of DMSO in culture medium. The stimulated cells were lysed in western and IP buffer (Beyotime, Jiangsu, China) and centrifuged at 4°C. Correlative proteins were immunoprecipitated from 1 mg MM cell lysate using IκBα (1:1000) or IgG (1:1000) antibody (rabbit) and coupled to protein A/G agarose beads (Beyotime, Jiangsu, China) overnight at 4 °C. The beads were washed with IP buffer, and the elution was processed by ubiquitin or IκBα immunoblotting.

### NF-κB inhibition assay

To determine the inhibition of NF-κB signaling, the transfected NF-κB/luciferase 293T cells were pre-treated with various concentrations of NNU219 or bortezomib for 6 h and then stimulated with 10 ng/ml of tumor necrosis factor-α (TNF-α) for 18 h, followed by measurement of luciferase activity by using the Dual-Luciferase^®^ Reporter Assay System (Promega Corp., Madison, WI, USA) as previously described [46].

### Real-time quantitative reverse transcriptase-polymerase chain reaction (RQ-PCR)

Total RNA was extracted using TRIzol reagent (Invitrogen; Thermo Fisher Scientific, Inc., USA). Real-time qPCR was performed using the RevertAid First Strand cDNA Synthesis Kit (Thermo Fisher Scientific, Inc., Waltham, MA, USA) and FastStart Universal SYBR Green Master (Rox; Roche) according to the manufacturer’s protocols. PCR reaction was performed in a 25-mL volume, and the thermocycling conditions were 95 °C for 30 min followed by 40 cycles of 95 °C for 60 sec and 60 °C for 20 sec. The following are the primer sequences: H-ICAM1-S, 5'-CCGTTGCCTAAAAAGGAGTTGC-3'; H-ICAM1-A, 5'-TGGCAGCGTAGGGTAAGGTTC-3';5'-H-TNF-α-STCTACTCCCAGGTCCTCTTCAAG-3';5'-H-TNF-α-AGGAAGACCCCTCCCAGATAGA-3';5'-H-IL-6-S, GTAGTGAGGAACAAGCCAGAGC-3'; 5'-H-IL-6-A, TACATTTGCCGAAGAGCCCT-3'; 5'-H-VEGF-S, GGAGGGCAGAATCATCACGA-3'; 5'-H-VEGF-AGCTCATCTCTCCTATGTGCTGG-3'. All PCRs were run in triplicate, and the mRNA levels of target genes relative to β-actin were calculated using the 2^-ΔΔCT^ method [48].

### Microsomal stability assay

Microsomal stabilities were determined by incubating 1 μM of the test compounds with rat, mouse, dog, monkey or human liver microsomes (Xenotech, USA) for different durations at 37 °C. Clearance and T_1/2_ were calculated by measuring the remaining percentage of test compounds at different time-points. Liquid chromatography/mass spectrometry (Xevo Quadrupole Time-of-Flight Mass Spectrometer; Waters) and an ACQUITY High-Performance Liquid Chromatography (HPLC) system (Shimadzu, Japan) were used to determine the relative peak area for the parent compounds.

### Pharmacodynamic study

For the pharmacodynamic study, female BNX mice (n=6; 5-7 weeks old; Shanghai Sippr-BK laboratory Animal Co. Ltd.) were given one single dose of NNU219 (0.4 mg/kg) and bortezomib (1 mg/kg) by i.v. administrations or NNU546 (2-15 mg/kg) by oral gavage. At different time-points after drug administrations, 100 μL of whole blood was collected from each mouse and processed for the 20S proteasome activity assay. After 1 or 24 h post-drug administration, tissue samples (heart, liver, spleen, lung, brain and tumors) were collected and homogenized in PBS buffer with three volumes of lysis buffer. Tissue homogenates and cell lysates were removed by centrifugation and supernatants were collected for protein quantitation and proteasome activity determination using the Proteasome-Glo assay reagents as described above [45].

### *In vivo* activity of NNU219 and NNU546 in ARH77 MM xenograft models

All experiments were performed according to protocols approved by the Institutional Animal Care and Use Committee of Nanjing Normal University. All animals were housed for 1-2 weeks prior to the experiment and maintained in a controlled environment with free access to food and water *ad libitum*.

For the ARH77 xenograft model, female BNX mice (n=5; 5-7 weeks old; Shanghai Sippr-BK laboratory Animal Co. Ltd.) were implanted with 5×10^6^ of ARH77 cells in Matrigel (1:1) and randomized into treatment and vehicle groups when the mean tumor volume (MTV) reached 100-150 mm^3^. For the intravenous treatment groups, mice were treated with bortezomib (1 mg/kg, BIW) or NNU219 (0.4 mg/kg, BIW), respectively. For i.g. administration, mice were treated with ixazomib (5 mg/kg, BIW) and NNU546 (1 mg/kg, QD) or NNU546 (2 mg/kg, QOD). Tumors were measured twice a week and the tumor volume was calculated using the following formula: 0.5 × (length × width^2^). Animals were euthanized when the tumors reached 2 cm^3^ or exhibited visible signs of necrosis.

### *In vivo* activity of NNU546 in PDX model

The PDX model was generated from biopsies of patients with MM brain manifestation at the Department of Hematology, the 1^st^ Affiliated Hospital of Nanjing Medical University. Freshly excised tumor tissue specimens were washed and cut into 2.5×2.5×2.5 mm^3^ pieces in antibiotic-containing L-15 medium. Under anesthesia with pentobarbital, 4-6 weeks-old male NCG mice (n=6; Model Animal Research Center of Nanjing University) were subcutaneously transplanted with the tumor pieces. Tumors were harvested when their size reached 500 mm^3^ (Px1 xenografts). Tumor tissue from Px1 xenograft mice was divided into small pieces and then implanted again subcutaneously as described above to obtain Px2 xenografts. This process was once more and the experiments were performed on Px3 xenograft mice, which were randomized into treatment groups when tumor volume reached 100-150 mm^3^. Mice were orally administered with ixazomib (5 mg/kg, i.g., BIW) or NNU546 (1 mg/kg, QD). Tumors were measured twice a week.

### *In vivo* activity of NNU546 in a SCID-rab model

For the SCID-rab model, RPMI 8226 cells were transfected with luciferase-expressing lentivirus and designated as RPMI 8226-Luc, which were then injected into rabbit bone fragments implanted subcutaneously into SCID mice (Shanghai Sippr-BK Laboratory Animal Co. Ltd.) as previously described [49]. The tumor burden was determined by monitoring the fluorescence activity with an IVIS 200 imaging system (Xenogen) [34]. Mice were orally administered with ixazomib (5 mg/kg, BIW) or NNU546 (1 mg/kg, QD).

For the construction of the primary myelomatous SCID-rab mouse model, unseparated myeloma BM cells from patients were injected directly into rabbit bone implanted subcutaneously into SCID mice (Shanghai Sippr-BK Laboratory Animal Co. Ltd.). The oral administrations of ixazomib and NNU546 were identical to those in the RPMI 8226-Luc SCID-rab model. Tumor growth was determined by the IgG levels in the blood of the mice using enzyme-linked immunosorbent assay (ELISA; FCMZCS, Nanjing, China) [49]. At the end of the experiment, bone grafts were radiated in 5-Gy fractions (total dose, 40 Gy) using a Faxitron CP-160 X-ray generating system (Faxitron X-Ray Corp., Wheeling, IL) at a dose rate of 0.5-1.5 Gy/min with 100 kVp and 10 mA. The grafts were gently extended into the radiation field with the remainder of the body of the anesthetized mouse shielded by custom-made cerrobend blocks [39]. Changes in bone mineral density (BMD) of the implanted bone and the femur of the mice were assessed using a microcomputed tomography (microCT) system (μCT 40; SKYSCAN). The bone grafts were scanned for qualitative assessment of the microarchitectureon μCT 40 using the manufacturer's software (CTan 1.10, Skyscan). Horizontally sectioned images were obtained with a voxel size of 9 μm. Semi-automated contouring was used to select a region of interest extending through the whole length of the grafts, composed of 100 adjacent individual two-dimensional slices. Analysis of the trabecular microarchitecture in the distal femur was performed using CTan program 1.10 [40].

### Histology and immunohistochemistry (IHC)

At the end of the *in vivo* activity experiments, mice were sacrificed, and tumors were immediately fixed in 10% of formalin for 24 h, and were then washed, dehydrated and embedded in paraffin. The bones were fixed, decalcified and embedded in paraffin. Finally, staining with hematoxylin-eosin (H&E) was performed using tumor or bone sections which were mounted on poly-lysine slides. On the hand, the main process of IHC is as follows: endogenous peroxidase activity was blocked with 3% (v/v) H_2_O_2_ for 15 min. After blocking with 5% BSA at 37°C for 0.5 h, the antigen was thermally repaired with citrate. The purified 14-3-3τ monoclonal antibody (Proteintech Group, Chicago, USA) was incubated overnight at 4°C. Afterwards, using secondary antibody for 0.75h at 37°C, followed by dropping SABC at 37°C for 30 min, DAB coloring, and finally counterstaining with hematoxylin. The histologic and IHC images were obtained by light microscopy using an optical microscope Nikon i55 (Nikon Corp., Tokyo, Japan) and analyzed.

### Immunofluorescent (IF) analysis of apoptosis and angiogenesis

The tumor sections obtained from xenograft mice described above were subjected to IF staining for Ki-67 or terminal deoxynucleotidyl transferase deoxyuridinetriphosphate nick end labelling (TUNEL). Tumor angiogenesis was assessed by IF staining for CD31 and α-smooth muscle actin (SMA) [31]. Microscopic images were captured using an UltraVIEW Vox confocal microscope (PerkinElmer, USA). 10 view fields were randomly selected and the fluorescence intensity was analyzed using image J software, and then the average value was calculated.

### Statistical analysis

Values were expressed as the mean ± standard deviation (SD) for at least two independent experiments. The two-tailed Student t-test and one-way ANOVA were used for comparisons of two or more than two groups, respectively. Statistical significance was defined as P<0.05. Statistical analysis was performed using GraphPad Prism 5 (GraphPad Software, Inc.).

## Supplementary Material

Supplementary Figures

Supplementary Tables
