# Supplementary material for: In vitro and in vivo efficacy of the novel oral proteasome inhibitor NNU546 in multiple myeloma
Source: Aging (Albany NY). 2020 Nov 16;12(22):22949–74. doi: 10.18632/aging.104023 (PMC7746380; doi:10.18632/aging.104023)
Supplement: Supplementary Figures [file aging-12-104023-s001..pdf]

SUPPLEMENTARY TABLES

Supplementary Table 1. The microsomal stabilities of the compounds.

Compound	Human liver microsomes		Rat liver microsomes		Mouse liver microsomes	
	$t_{1/2}$ (min)	CL_{int} (mL/min/kg)	$t_{1/2}$ (min)	CL_{int} (mL/min/kg)	$t_{1/2}$ (min)	CLint (mL/min/kg)
Bortezomib	21.4	58.3	12.4	200.5	10.7	508.7
NNU219	32.4	43	27.8	50	16.7	83

Note: The microsomal stabilities of bortezomib and NNU219 were determined in the presence of pooled human, rat or mouse liver microsomes. $T_{1/2}$ (min) and CL_{int} (mL/min/kg) were detected by LC/MS. Antipyrine and testosterone (5 µM each) were used as positive and negative controls, respectively. Abbreviations: t = time; CL_{int}, intrinsic clearance.

Supplementary Table 2. Pharmacokinetic profiles of NNU546 in mice.

	$t_{1/2}$ (h)	CL ($mL \cdot h^{-1} \cdot kg^{-1}$)	Vz ($mL \cdot kg^{-1}$)	$AUC_{0\text{-}t}$ ($h \cdot ng \cdot mL^{-1}$)	MRT (h)	F(%)
i.v.	2.08±0.991	986±75.4	2914±1247	1890±73.8	1.55±0.133	---
i.g.	2.41±0.420	---	---	208±26.7	2.35±0.221	11.0±1.41

Note: Blood samples were collected at baseline and after intravenous or oral administration of 2 mg/kg of NNU546. Each time-point represents the average value of three animals. NNU546 concentration in blood and plasma samples was determined by liquid chromatography-tandem mass spectrometry (LC/MS/MS) in a non–good laboratory practice lab. Pharmacokinetic analysis of the blood and plasma concentration data was performed using WinNonlin version 5.2 (Pharsight Corp.). Kinetic parameters were estimated using a noncompartmental model with sparse sampling mode (model 201 for plasma and blood). Area under the concentration vs. time curve (AUC) was calculated using the linear trapezoidal rule. Abbreviations: t = time; CL, clearance; Vz, apparent volume of distribution; $AUC_{0–t}$, AUC (area under the curve) from 0 to t h; MRT, mean residence time; F%, oral bioavailability.
