# Supplementary material for: In vitro and in vivo efficacy of the novel oral proteasome inhibitor NNU546 in multiple myeloma
Source: Aging (Albany NY). 2020 Nov 16;12(22):22949–74. doi: 10.18632/aging.104023 (PMC7746380; doi:10.18632/aging.104023)
Supplement: Supplementary Tables [file aging-12-104023-s002..pdf]

SUPPLEMENTARY FIGURES

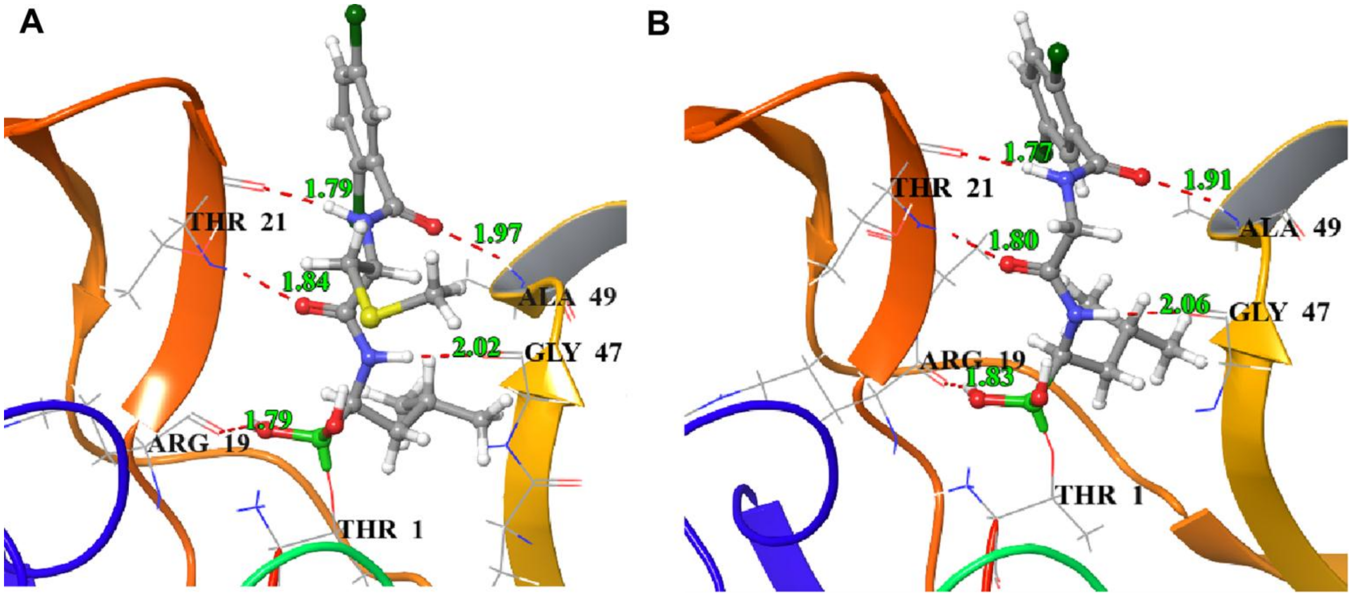

Supplementary Figure 1. Covalent docking with β5 subunit. (**A**) Binding mode of NNU219 with β5 subunit; (**B**) Binding mode of MLN2238 with β5 subunit. All the hydrogen atoms were not shown. The amino acid residues of β5 subunit were shown in ribbon models. NNU219 and MLN2238 were represented as ball and stick models. The pictures were rendered in PyMOL.

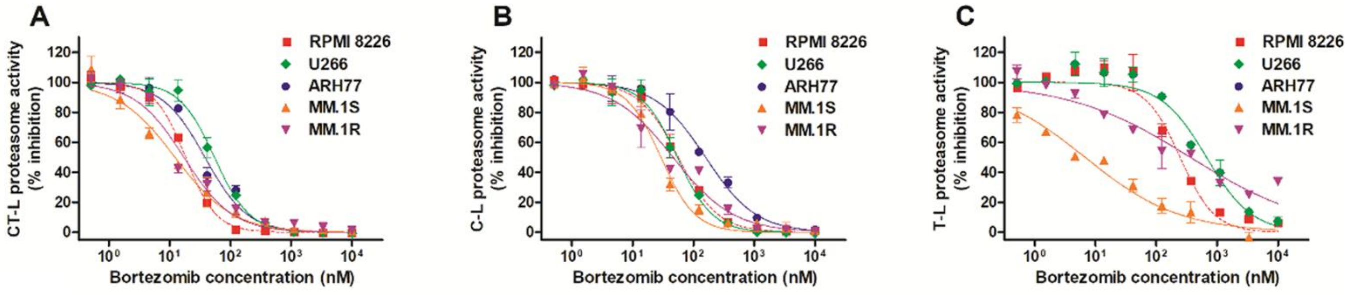

Supplementary Figure 2. Proteasome active site selectivity of bortezomib in human MM cell lines. MM cells were treated with various concentrations of bortezomib for 1 h and harvested and cytosolic extracts were then analyzed for CT-L (**A**), C-L (**B**) and T-L (**C**) proteasome activity. Results were represented as percent activity of proteasome in drug-treated cells vs. vehicle-treated cells (±SD).

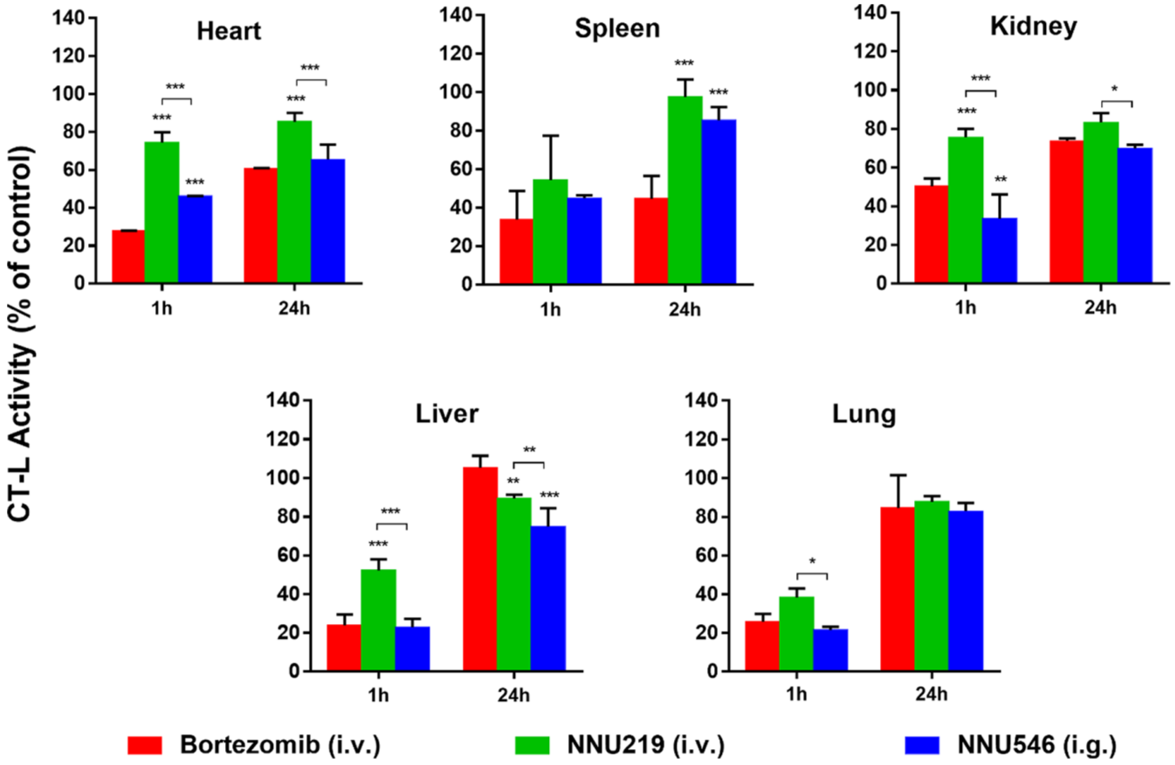

Supplementary Figure 3. Pharmacodynamic profiles of proteasome inhibition in mice bearing human multiple myeloma xenografts. ARH77 tumor-bearing mice were administered with a single dose of NNU219 (0.4 mg/kg, i.v.), NNU546 (2 mg/kg, i.g.) or bortezomib (1 mg/kg, i.v.) and were euthanized at 1 h and 24 h after administrations. Blood and normal tissues (spleen, kidney, liver, lung) were harvested and then protein extracts were prepared and the proteasome catalytic activity was evaluated with CT-L subunit-specific fluorescent peptide substrates. Data were presented as the mean ±SD percent activity relative to vehicle controls (3 mice per time-point). *p* values presented for bortezomib vs NNU219 or NNU546 (*, $p < 0.5$; **, $p < 0.01$; ***, $p < 0.001$).

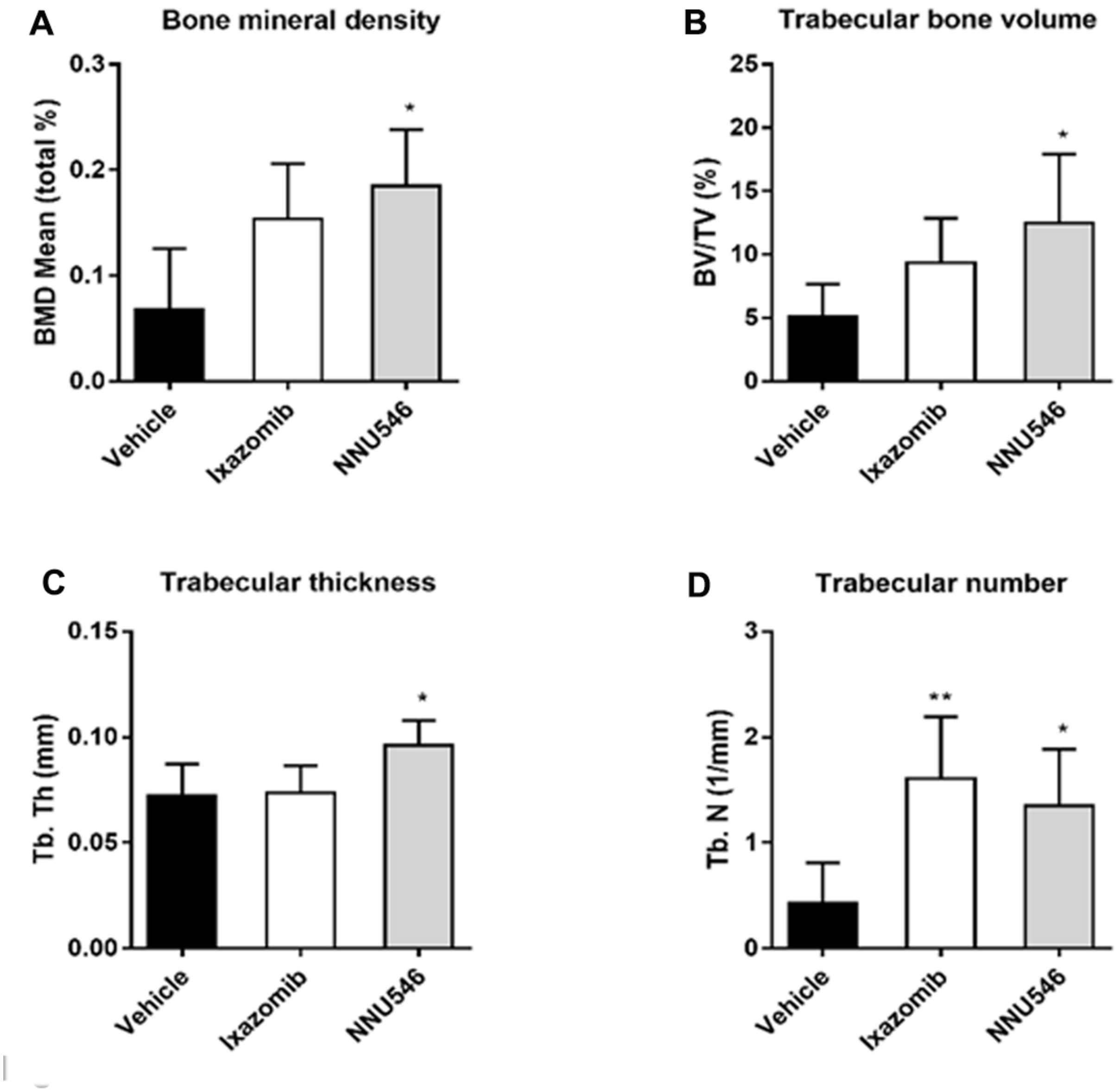

Supplementary Figure 4. NNU546 promoted bone formation in myelomatous bones. SCID-rab mice engrafted with myeloma cells of MM patients were treated with vehicle, ixazomib (5 mg/kg, BIW) or NNU546 (1 mg/kg, QD). Levels of BMD (**A**) and static histomorphometry (**B**–**D**) of the implanted bones at the end of experiment. Data were presented as mean ± SD (n=5; *, $p < 0.05$; **, $p < 0.01$). Y-axis abbreviation: BMD, bone mineral density; BV, bone volume; TV, tissue volume; Tb, trabecular; Th, thickness; N, number.
